# Functional informed genome‐wide interaction analysis of body mass index, diabetes and colorectal cancer risk

**DOI:** 10.1002/cam4.2971

**Published:** 2020-03-24

**Authors:** Zhiyu Xia, Yu‐Ru Su, Paneen Petersen, Lihong Qi, Andre E. Kim, Jane C. Figueiredo, Yi Lin, Hongmei Nan, Lori C. Sakoda, Demetrius Albanes, Sonja I. Berndt, Stéphane Bézieau, Stephanie Bien, Daniel D. Buchanan, Graham Casey, Andrew T. Chan, David V. Conti, David A. Drew, Steven J. Gallinger, W. James Gauderman, Graham G. Giles, Stephen B. Gruber, Marc J. Gunter, Michael Hoffmeister, Mark A. Jenkins, Amit D. Joshi, Loic Le Marchand, Juan P. Lewinger, Li Li, Noralane M. Lindor, Victor Moreno, Neil Murphy, Rami Nassir, Polly A. Newcomb, Shuji Ogino, Gad Rennert, Mingyang Song, Xiaoliang Wang, Alicja Wolk, Michael O. Woods, Hermann Brenner, Emily White, Martha L. Slattery, Edward L. Giovannucci, Jenny Chang‐Claude, Paul D. P. Pharoah, Li Hsu, Peter T. Campbell, Ulrike Peters

**Affiliations:** ^1^ Department of Epidemiology University of Washington Seattle WA USA; ^2^ Public Health Sciences Fred Hutchinson Cancer Research Center Seattle WA USA; ^3^ Department of Public Health Sciences University of California Davis Davis CA USA; ^4^ Department of Preventive Medicine & USC Norris Comprehensive Cancer Center Keck School of Medicine University of Southern California Los Angeles CA USA; ^5^ Department of Medicine Samuel Oschin Comprehensive Cancer Institute Cedars‐Sinai Medical Center Los Angeles CA USA; ^6^ Department of Epidemiology Richard M. Fairbanks School of Public Health Indiana University Indianapolis IN USA; ^7^ Division of Research Kaiser Permanente Northern California Oakland CA USA; ^8^ Division of Cancer Epidemiology and Genetics National Cancer Institute National Institutes of Health Bethesda MD USA; ^9^ Service de Génétique Médicale Centre Hospitalier Universitaire (CHU) Nantes Nantes France; ^10^ Colorectal Oncogenomics Group Department of Clinical Pathology The University of Melbourne Parkville Victoria Australia; ^11^ University of Melbourne Centre for Cancer Research Victorian Comprehensive Cancer Centre Parkville Victoria Australia; ^12^ Genetic Medicine and Family Cancer Clinic The Royal Melbourne Hospital Parkville Victoria Australia; ^13^ Center for Public Health Genomics University of Virginia Charlottesville VA USA; ^14^ Division of Gastroenterology Massachusetts General Hospital Harvard Medical School Boston MA USA; ^15^ Channing Division of Network Medicine Brigham and Women's Hospital Harvard Medical School Boston MA USA; ^16^ Clinical and Translational Epidemiology Unit Massachusetts General Hospital Harvard Medical School Boston MA USA; ^17^ Broad Institute of Harvard and MIT Cambridge MA USA; ^18^ Department of Epidemiology Harvard T.H. Chan School of Public Health Harvard University Boston MA USA; ^19^ Department of Immunology and Infectious Diseases Harvard T.H. Chan School of Public Health Harvard University Boston MA USA; ^20^ Lunenfeld Tanenbaum Research Institute Mount Sinai Hospital University of Toronto Toronto Ontario Canada; ^21^ Cancer Epidemiology Division Melbourne Victoria Australia; ^22^ Centre for Epidemiology and Biostatistics Melbourne School of Population and Global Health The University of Melbourne Melbourne Victoria Australia; ^23^ Section of Nutrition and Metabolism International Agency for Research on Cancer Lyon France; ^24^ Division of Clinical Epidemiology and Aging Research German Cancer Research Center (DKFZ) Heidelberg Germany; ^25^ University of Hawaii Cancer Center Honolulu HI USA; ^26^ Department of Family Medicine University of Virginia Charlottesville VA USA; ^27^ Department of Health Science Research Mayo Clinic Scottsdale AZ USA; ^28^ Oncology Data Analytics Program Catalan Institute of Oncology‐IDIBELL, L'Hospitalet de Llobregat Barcelona Spain; ^29^ CIBER Epidemiología y Salud Pública (CIBERESP) Madrid Spain; ^30^ Department of Clinical Sciences Faculty of Medicine University of Barcelona Barcelona Spain; ^31^ ONCOBEL Program Bellvitge Biomedical Research Institute (IDIBELL) L'Hospitalet de Llobregat Barcelona Spain; ^32^ Department of Pathology School of Medicine Umm Al‐Qura University Makkah Saudi Arabia; ^33^ Department of Pathology Brigham and Women's Hospital Harvard Medical School Boston MA USA; ^34^ Department of Oncologic Pathology Dana‐Farber Cancer Institute Boston MA USA; ^35^ Department of Community Medicine and Epidemiology Lady Davis Carmel Medical Center Haifa Israel; ^36^ Ruth and Bruce Rappaport Faculty of Medicine Technion‐Israel Institute of Technology Haifa Israel; ^37^ Clalit National Cancer Control Center Haifa Israel; ^38^ Department of Nutrition Harvard T.H. Chan School of Public Health Harvard University Boston MA USA; ^39^ Institute of Environmental Medicine Karolinska Institutet Stockholm Sweden; ^40^ Memorial University of Newfoundland Discipline of Genetics St. John's Canada; ^41^ Division of Preventive Oncology German Cancer Research Center (DKFZ) National Center for Tumor Diseases (NCT) Heidelberg Germany; ^42^ Department of Internal Medicine University of Utah Salt Lake City UT USA; ^43^ Division of Cancer Epidemiology German Cancer Research Center (DKFZ) Heidelberg Germany; ^44^ University Medical Centre Hamburg‐Eppendorf University Cancer Centre Hamburg (UCCH) Hamburg Germany; ^45^ Department of Public Health and Primary Care University of Cambridge Cambridge UK; ^46^ Department of Biostatistics University of Washington Seattle WA USA; ^47^ Behavioral and Epidemiology Research Group American Cancer Society Atlanta GA USA

**Keywords:** BMI, colorectal cancer, diabetes, gene expression, gene‐environmental interaction

## Abstract

**Background:**

Body mass index (BMI) and diabetes are established risk factors for colorectal cancer (CRC), likely through perturbations in metabolic traits (e.g. insulin resistance and glucose homeostasis). Identification of interactions between variation in genes and these metabolic risk factors may identify novel biologic insights into CRC etiology.

**Methods:**

To improve statistical power and interpretation for gene‐environment interaction (G × E) testing, we tested genetic variants that regulate expression of a gene together for interaction with BMI (kg/m^2^) and diabetes on CRC risk among 26 017 cases and 20 692 controls. Each variant was weighted based on PrediXcan analysis of gene expression data from colon tissue generated in the Genotype‐Tissue Expression Project for all genes with heritability ≥1%. We used a mixed‐effects model to jointly measure the G × E interaction in a gene by partitioning the interactions into the predicted gene expression levels (fixed effects), and residual G × E effects (random effects). G × BMI analyses were stratified by sex as BMI‐CRC associations differ by sex. We used false discovery rates to account for multiple comparisons and reported all results with FDR <0.2.

**Results:**

Among 4839 genes tested, genetically predicted expressions of *FOXA1* (*P* = 3.15 × 10^−5^), *PSMC5* (*P* = 4.51 × 10^−4^) and *CD33* (*P* = 2.71 × 10^−4^) modified the association of BMI on CRC risk for men; *KIAA0753* (*P* = 2.29 × 10^−5^) and *SCN1B* (*P* = 2.76 × 10^−4^) modified the association of BMI on CRC risk for women; and *PTPN2* modified the association between diabetes and CRC risk in both sexes (*P* = 2.31 × 10^−5^).

**Conclusions:**

Aggregating G × E interactions and incorporating functional information, we discovered novel genes that may interact with BMI and diabetes on CRC risk.

## Graphical abstract

Novel findings of this study include: the genetically predicted expressions of FOXA1, PSMC5 and CD33 modified the association of BMI on CRC risk for men; KIAA0753 and SCN1B modified the association of BMI on CRC risk for women; and PTPN2 modified the association between diabetes and CRC risk in both sexes. These findings provide support for potential new biological insights that could help in understanding the underlying mechanisms of BMI and diabetes on CRC.

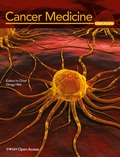

## 1 BACKGROUND

Colorectal cancer (CRC) is a major source of cancer morbidity and mortality worldwide. According to the World Health Organization (WHO), CRC is the third most common cancer worldwide and accounts for approximately 10% of global cancer incidence and mortality (http://globocan.iarc.fr/). Genetic factors play an important role in the etiology of both familial and sporadic CRC.^1^ CRC is a complex, multifactorial disease with many genetic and modifiable lifestyle factors including diet,^2^ obesity,^3^ physical activity,^4^ and diabetes^5^ among others contributing to its etiology.

Obesity, compared to normal weight, is associated with greater risk of CRC^6^ in a dose‐response manner.^3^ According to a recent World Health Organization report, 39% of adults (1.9 billion) aged 18 years and older were overweight, and 13% (650 million) were obese (https://www.who.int/news-room/fact-sheets/detail/obesity-and-overweight). With a growing obesity epidemic, the number of people with diabetes has also dramatically increased from 108 million in 1980 to around 500 million in 2018 worldwide (https://www.who.int/news-room/fact-sheets/detail/diabetes). These trends are expected to continue, and will likely continue to contribute to the burden imposed by CRC in the coming decades.^7^ Sex has been found to modify the association between obesity and CRC risk: the risk of developing CRC is often higher in obese men compared to obese women.^8^ These sex differences may be contributed to differences in sex hormones whereby estrogens, in particular, derived from adipose tissue offset the risk otherwise mitigated by obesity more so for women than men.^9^ Obesity and diabetes are interrelated risk factors for CRC that may impact CRC risk through metabolic abnormalities including pathways related to inflammation, insulin, and glucose homeostasis.^10^ Findings from meta‐analyses have indicated that diabetes is associated with an approximately 30% increased relative risk of developing colorectal cancer compared to nondiabetes, after adjusting for BMI.^5^

It has been estimated that genetic variants explain up to 35% of the heritability in CRC risk.^11^ To date, genome‐wide association studies (GWAS) have identified more than 100 independent common genetic variants that are robustly associated with CRC.^12^ However, these variants only explain a fraction of total heritability. Given the complexity of CRC etiology, it can be expected that a closer investigation of gene‐environment (G × E) interactions may help identify additional novel loci and biological interactions that give insight to the pathogenesis of CRC. A few studies have investigated G × E interactions with BMI and diabetes for CRC risk, mainly focusing on the single nucleotide polymorphisms (SNPs) that had been previously identified by GWAS.^13^, ^14^ The candidate G × E analysis by Sainz et al indicated that SNPs in *IGF2BP2* (rs4402960) and *PPARγ* (rs1801282) may interact with diabetes on CRC risk.^15^ As statistical power remains a major concern for G × E analysis, conducting set‐based G × E testing and incorporating functional genomic information may improve power and help to interpret the underlying biology.

In this study, we conducted a novel set‐based genome‐wide approach to test interactions between genetic predicted gene expression and BMI and diabetes with CRC risk. We applied MiSTi, a set‐based G × E testing framework which allows for incorporation of functional information.^16^

## 2 METHODS

### 2.1 Study participants

We used epidemiological and genetic data of 26 017 incident CRC cases and 20 692 controls from 33 participating studies in three international CRC consortia: the Genetics and Epidemiology of Colorectal Cancer Consortium (GECCO), the Colorectal Transdisciplinary Study (CORECT) and the Colon Cancer Family Registry (CCFR). Details have been published previously.^13^, ^17^ Participants with non‐European ancestry were excluded because of small sample sizes. All studies were approved by their respective Institutional Review Boards.

### 2.2 Genotype data

Details on genotyping and imputation have been reported previously.^17^, ^18^ In brief, DNA was mostly obtained from blood/buccal samples. Several platforms (the Illumina HumanHap 300 k, 240 k, 550 k and OncoArray 610 k BeadChip Array system, or Affymetrix platform) were used for genotyping.^19^ Samples were excluded based on sample call rates ≤97%, heterozygosity, unexpected duplicates or relative pairs, gender discrepancy and principal component analysis (PCA) outlier of HapMap2 CEU cluster. SNPs were excluded on the basis of inconsistency across platforms, call rate <98%, and out of Hardy‐Weinberg equilibrium (HWE) in controls (*P* < .0001).^19^ SNPs were imputed to the Haplotype Reference Consortium (HRC version r1.0),^20^ and restricted by imputation accuracy (*R* ^2^ > 0.3 for SNPs with MAF >1%, *R* ^2^ > 0.5 for SNPs with MAF >0.5% and <1%, and *R* ^2^ > 0.99 for SNPs with MAF <0.05%).

### 2.3 Estimation of gene expression levels

Functional information was generated from PrediXcan,^21^ which used an elastic net penalized regression to select eQTLs that jointly predict gene expression levels based on genome‐wide genotypes and transcriptome data from 169 colon tissue samples from the GTEx project (GTEx v6). While GTEx measured gene expression at two different locations in the colon (sigmoid and transverse), we restricted analysis to the transverse. The transverse colon samples were done on the entire colonic wall while the analysis of the sigmoid colon was restricted to the muscularis tissue, which is less relevant for CRC development. The heritability of gene expression levels explained by the SNPs (predictive *R* ^2^) were calculated using a mixed‐effects model.^21^, ^22^ The estimated weights and predictive *R* ^2^ for eQTL sets associated with individual genes were downloaded from the publicly available PredictDB Repository (http://hakyimlab.org/predictdb/). Genes with *R* ^2^ ≥ 0.01 were selected for interaction analyses. A total of 4839 genes were included.

### 2.4 Exposure assessment

Demographics and environmental exposures were self‐reported at either in‐person interview or via structured self‐administered questionnaires, based on each participating study. A multistep, iterative data harmonization procedure was applied, reconciling each study's unique protocols and data collection instruments.^13^, ^23^ Numerous quality‐control checks were performed, and outlying values of variables were truncated to the minimum or maximum value of an established range for each variable. Variables were combined into a single dataset with common definition, standardized coding, and standardized permissible values. For the main exposure variables (BMI and diabetes), continuous measurement of BMI (per 5 kg/m^2^, excluded participants below 18.5 kg/m^2^) as well as a binary self‐reported diagnosis of diabetes were used.

### 2.5 Statistical analysis

Individual level genotyping and environmental data were used for statistical analysis. We used MiSTi,^16^ a set‐based statistical framework providing mixed effects score tests for G × E interaction, to identify genes with eQTLs that interact with BMI or diabetes on CRC risk. MiSTi models the G × E interaction effects with two components, the fixed‐ and random‐effect components. The fixed‐effect component incorporates the weights from PrediXcan to calculate the genetically predicted gene expression levels for samples in our data, and then assesses the interaction between the predicted expression of each gene and BMI as well as diabetes. The random‐effects component quantifies residual interaction effects that are not accounted for by the fixed‐effect component. To combine the fixed and random effects components, we used the data‐adaptive weighted combination approach under MiSTi (aMiSTi).^16^ All BMI analyses were stratified by sex as BMI‐CRC associations differ by sex. As associations for diabetes and CRC have been reported to be similar for men and women, we analyzed diabetes‐by‐SNP associations in men and women combined and included sex as a covariable. Genes with false discovery rate (FDR) <0.2 were considered statistically significant.

For G × E interactions discovered in our analysis, we conducted secondary analyses with multivariable‐adjusted generalized linear regression models to assess main effects and interactions between individual eQTL variants and BMI/diabetes on CRC risk. A sequential analysis was conducted where we started with the most significant SNP with the G × E interaction, then took the next significant one while adjusting for the first one, and so forth until the SNP’s *P*‐value was greater than .05. Age, study and PCs were adjusted for BMI models, and sex was additionally included in the sequential analysis for diabetes‐by‐SNP associations. eQTL variants which drive the significant interaction effects were identified and reported. All statistical analyses were performed using R (version 3.4.4).

## 3 RESULTS

The demographic characteristics and exposures of interest are summarized in Table S1. Compared to controls, cases had a higher BMI (27.4 vs 26.7 kg/m^2^) and a higher prevalence of diabetes (13.5% vs 10.7%). The main effects of BMI and diabetes on CRC risk for each study individually and combined are summarized in Figures [Link], [Link]A, S1B, and S2, respectively. Among men, each 5 kg/m^2^ increase in BMI was associated with 24% higher risk of CRC (fixed effect OR = 1.24, 95% CI 1.19‐1.29, *P*‐value < .01); among women, each 5 kg/m^2^ increase in BMI was associated with 12% higher risk of CRC (fixed effect OR = 1.12, 95% CI 1.09‐1.15, *P*‐value < .01). In addition, diabetes was associated with a 22% higher risk of CRC (fixed effect OR = 1.22, 95% CI 1.14‐1.30, *P*‐value < .01) compared to those without diabetes.

Among the 4839 genes tested, we observed interactions between genetically predicted expression and BMI for CRC risk at FDR <0.2 for three genes among men, and two genes among women (Table 1; Figure [Link], [Link]A and S3B). All genetic signals in the five genes were detected by the random‐effect components (Table 1). Among men, the most significant gene was *FOXA1* (p_interaction_ = 3.15 × 10^−5^) located on 14q21.1. A total of 43 eQTLs were included in this gene. The second most significant gene was *CD33* (p_interaction_ = 2.71 × 10^−4^, located on 19q13.3) with 30 eQTL included in the interaction test for this gene. Another gene, *PSMC5*, located on 17q23.3 also surpassed the FDR threshold and interacted with BMI among men (p_interaction_ = 4.51 × 10^−4^), with 32 eQTL tested. Among women, *KIAA0753* (p_interaction_ = 2.29 × 10^−5^, located on 17p13.1) and *SCN1B* (p_interaction_ = 2.76 × 10^−4^, located on 19q13.11) showed interactions with BMI on CRC risk. There were 30 and 11 eQTLs included in the interaction testing of these two genes, respectively.

**Table 1:** Results for interactions of eQTLs with BMI and diabetes and risk of colorectal cancer with a FDR <0.2

Gene name	CHR	# of SNPs	^a^ *R* ^2^	*P*‐values		
				Fixed‐effect component	Random‐effect component	Adaptive weight
BMI						
Male						
*FOXA1*	14q21.1	43	1.90%	0.125	1.03 × 10^−5^	3.15 × 10^−5^
*CD33*	19q13.3	30	1.87%	0.963	1.28 × 10^−4^	2.71 × 10^−4^
*PSMC5*	17q23.3	32	10.50%	0.719	9.96 × 10^−5^	4.51 × 10^−4^
Female						
*KIAA0753*	17p13.1	30	14.49%	0.029	5.91 × 10^−5^	2.29 × 10^−5^
*SCN1B*	19q13.11	11	2.27%	0.426	6.33 × 10^−5^	2.76 × 10^−4^
Diabetes						
*PTPN2*	18p11.21	95	7.87%	1.03 × 10^−5^	0.605	2.31 × 10^−5^

^a^ *R* ^2^ is the heritability in gene expression.

When studying interactions with diabetes, we observed that the eQTLs of *PTPN2* located at 18p11.21 modified the association between diabetes and CRC risk (p_interaction_ = 2.31 × 10^−5^) (Table 1; Figure S4). The interaction signal was mainly from the interaction of genetically predicted gene expression with diabetes (p_interaction_ = 1.03 × 10^−5^), detected by the fixed‐effect components. A total of 95 eQTLs predicted the expression level of this gene.

In secondary analysis we assessed main effects and interactions of individual variants with BMI or diabetes on CRC risk for each gene set that showed FDR <0.2. The results suggested that associations observed for *FOXA1*, *CD33*, *PSMC5*, and *PTPN2* might be largely driven by some specific individual variants (Table S2). In Tables S3A and S3B, we demonstrated the associations of the predicted gene expression on CRC risk stratified by BMI at quartiles and diabetes.

## 4 DISCUSSION

In this large genome‐wide investigation using transcription data to inform G × E interaction testing, we observed suggested interactions between genetic predicted gene expression and BMI and diabetes for risk of CRC. The strongest association was for *FOXA1* and BMI with CRC risk for men, and the predicted gene expression level of *PTPN2* interacted with diabetes on CRC risk among men and women combined. Our findings also suggested that the gene expression levels of *CD33* and *PSMC5* may interact with BMI on CRC risk among men, and the gene expression levels of *KIAA0753* and *SCN1B* may interact with BMI on CRC risk among women. The identified genes are novel in modifying BMI‐CRC and diabetes‐CRC associations.

Our most significant result in the BMI interaction analysis was for the *FOXA1* (Forkhead Box A1) gene‐BMI interaction for CRC risk, among men. *FOXA1* is a transcription factor that belongs to the *FOX* gene superfamily,^24^ which is responsible for various biological processes, including cell proliferation, apoptosis and differentiation.^25^ Several studies have found that *FOXA1* expression in cancer tissues was associated with multiple types of human cancers,^26^, ^27^ reflecting its crucial roles in cellular processes. According to Sahu et al,^27^ besides pioneering the androgen receptor (AR) pathway, *FOXA1* depletion elicited extensive redistribution of AR‐binding sites on LNCaP‐1F5 cell chromatin that was commensurate with changes in androgen‐dependent gene expression signature. They also found that the role of *FOXA1* in androgen signaling is distinctly different from that in estrogen signaling, providing evidence to the results that *FOXA1* was associated with CRC and interacted with BMI only among men, but not women. A recent study detected the expression of *FOXA1* in samples of CRC tissues and matched noncancerous tissues using immunohistochemistry to determine the clinical significance of *FOXA1* and its role in CRC.^28^ Their research demonstrated that the *FOXA1* expression level in cancer tissues was significantly higher among CRC cases compared to noncancer specimens, and positive expression of *FOXA1* in cancer tissues was associated with poor clinicopathological characteristics as well as poor prognosis of CRC.^28^ Though many existing studies indicated the strong associations between *FOXA1* expression in cancer tissues and human cancers, so far none of them focused on interactions between *FOXA1* and BMI on cancers. In our genome‐wide G × BMI interaction scans, we demonstrated that genetic variants in *FOXA1* interacted with BMI among men. Further exploration suggested that multiple genetic variants in the tested gene‐set contributed to the *FOXA1*‐BMI interaction effect. This interaction may be explained by the observation that *FOXA1* binds to four distinct intronic regions of the *FTO* (fat mass and obesity associated) gene,^29^ which has a known predisposing role to obesity.^30^ However, functional follow up analysis are needed to shed further light on this interaction effect of this gene. Overall, previous literature provides strong support for a potential role of *FOXA1* in CRC which may be mediated through the *FTO* gene that could explain the observed interaction with obesity.

We identified an interaction between *PTPN2* (protein tyrosine phosphatase nonreceptor‐type 2) and diabetes with CRC risk. *PTPN2* gene encodes the T‐cell‐specific protein tyrosine phosphatase, and functions as a negative regulator of inflammation by inhibiting the transcription factor *STAT1* in the *IFN‐γ* signaling pathway.^31^ Previous studies demonstrated that several variants located in *PTPN2* were significantly associated with inflammatory bowel disease,^32^ celiac disease,^33^ rheumatoid arthritis ^34^ and diabetes.^35^ A recent study identified the dual role for *PTPN2* in directly regulating inflammasome activation and IL‐1β production to suppress pro‐inflammatory responses during colitis but promote intestinal tumor development.^36^ Since diabetes is also an inflammation‐related disorder,^37^ it might suggest a mechanism on how *PTPN2* interacts with diabetes on CRC risk. *PTPN2* was also found to be associated with activation of PI3K/AKT pathway and tamoxifen resistance in breast cancer.^38^ PI3K/AKT is a well‐documented pathway associated with human cancer risk that heavily regulates glucose and IGF signaling^39^; therefore, it is possible that the expression of *PTPN2* interacts with diabetes through regulation of a variety of tyrosine kinases, given that tyrosine kinase activity of the insulin receptor is associated with human diabetes.^40^ Again, functional follow up will be needed to better understand the interaction of this strong candidate gene.

We also observed a suggestive interaction between the *CD33* gene and BMI on CRC risk among men. We identified two genetic variants that were likely at least in part driving the significance. The *CD33* gene encodes a differentiation antigen of acute myeloid leukemia (AML) progenitor cells and is a very well‐known pathological marker of AML.^41^ *CD33* is a transmembrane receptor, and was found to express on myeloid and lymphoid cells in about 85%‐90% of patients with acute myeloid leukemia.^42^ *CD33* gene expression was also discovered to inhibit Ca^2+^ flux, cell growth and apoptosis.^43^ Even though the association between *CD33* and AML were well‐studied, how *CD33* is associated with CRC risk and how it interacts with BMI on CRC risk is yet to be identified. Previous research indicated that the frequency of *CD33 + *cells in blood, as a subset of myeloid‑derived suppressor cells, was significantly higher in obese subjects compared to nonobese individuals,^44^ providing some evidence for a potential modifying effect of obesity. Our study provides preliminary results for a potential interaction between *CD33* and BMI on CRC risk that will require additional follow up analysis.

We identified that the *SCN1B* (Sodium Voltage‐Gated Channel Beta Subunit 1) gene interacted with BMI on CRC risk among women at FDR <0.2. Voltage‐gated sodium channels (Na_V_)^45^ are composed of one large pore‐forming principal subunit and one or two smaller transmembrane subunits considered as auxiliary, and the *SCN1B* gene generates one of such subunits.^45^ Multiple studies have demonstrated that its expression regulated cellular functions such as migration, differentiation, endosome acidification, phagocytosis, and podosome formation.^46^ In addition, Na_V_ are found abnormally expressed in carcinoma cells and tumor biopsies, and their activity is associated with aggressive features and cancer progression.^47^ Expression of the Na_V_1.5 isoform in breast tumors was found to be correlated with metastases development and patients’ death,^48^ and *SCN1B* mRNA was also discovered to be more abundant in highly invasive prostate cancer cell lines.^49^ Our study revealed that the gene *SCN1B* significantly interacted with BMI on CRC risk, though the interaction became nonsignificant after Bonferroni correction. It is possible that obesity interacts with Na_V_ and further affects the regulations of cellular functions, resulting in various types of human cancers including CRC; further follow up studies are warranted.

Several strengths and limitations need to be considered when interpreting the findings. In our study, which is the largest to date to investigate gene‐BMI and gene‐diabetes interactions on CRC risk, we integrated colon‐specific gene expression data to inform interaction testing. However, the tissue collection of the gene expression is suboptimal given that the transverse colon tissue samples from the GTEx Project covers the entire colonic wall, which not only included the epithelial cells of the mucosa from which CRC derived, but also all other tissue layers, which dilutes the epithelial gene expression profile. For this reason, we used the gene expression data from the transverse colon tissues instead of the sigmoid colon tissues in the GTEx project, as the sigmoid colon tissue samples were collected from the muscle tissues only. We did not include other tissues, as we have observed that colorectal cancer risk loci are enriched of colorectal tissue specific enhancer marks, which are key regulators for gene expression.^50^ We also did not narrow down our analysis further using marginal eQTLs that have achieved transcriptome‐wide association study (TWAS) significance level, because we were concerned that we might miss novel interactions. We combined colon and rectal cancer cases together, because the comprehensive analysis of The Cancer Genome Atlas (TCGA) demonstrated that colon and rectal cancer are very similar.^51^ To improve statistical power, we used our novel statistical set‐based G × E mixed effects score tests, MiSTi, which allows testing of both fixed‐ and random‐effects of the interaction. As expected, the predicted heritability in gene expression differs between the genes. Accordingly, our statistical power to detect interaction between genetic‐defined gene expression (fixed effects) varies and is higher for more heritable genes, given the effect size are the same. In other words, if there is little evidence for E and predicted gene expression, it could be due to low *R* ^2^ for the gene. Since expression levels of different genes may differ across populations and our analysis was limited to those of European descent, our results may not be necessarily generalizable to other race/ethnicity groups. Furthermore, the self‐reported BMI and diabetes measurements might be subjected to recall bias, however, we found similar effects for prospective cohort studies and case‐control studies. Lastly, future independent replications are warranted, since FDR <0.2 is not stringent.

In summary, by incorporating functional information and conducting aggregated gene‐based testing, the most significant interactions we observed where between *FOXA1* and BMI among men, and between *PTPN2* and diabetes for CRC risk. Other suggested genes interacting with BMI at FDR <0.2 included *CD33* and *PSMC5* among males, and *SCN1B* and *KIAA0753* among females. These findings provide support for potential new biological insights that could help in understanding the underlying mechanisms of BMI and diabetes on CRC. Independent replication and functional follow‐up studies are warranted to confirm the functions of these genes in relation to BMI/diabetes and CRC development.

## CONFLICT OF INTEREST

None.

## Funding Information

National Cancer Institute, National Institutes of Health, US Department of Health and Human Services (U01 CA164930, U01 CA137088, R01 CA059045, R01201407); Center for Inherited Disease Research (CIDR) (X01‐HG008596 and X‐01‐HG007585); NIH/NCI Cancer Center Support Grant P30 CA015704; Hospital Clinical Research Program (PHRC‐BRD09/C) from the University Hospital Center of Nantes (CHU de Nantes); Regional Council of Pays de la Loire, the Groupement des Entreprises Françaises dans la Lutte contre le Cancer (GEFLUC), the Association Anne de Bretagne Génétique and the Ligue Régionale Contre le Cancer (LRCC). Intramural Research Program of the US National Cancer Institute, National Institutes of Health, and by US Public Health Service contract HHSN261201500005C from the National Cancer Institute, Department of Health and Human Services. COLO2&3: National Institutes of Health (R01 CA60987); National Cancer Institute (NCI), National Institutes of Health (NIH) (grant numbers U01 CA122839, R01 CA143247); NCI/NIH (grant number U01 CA167551); Australasian Colorectal Cancer Family Registry (grant numbers U01 CA074778 and U01/U24 CA097735); USC Consortium Colorectal Cancer Family Registry (grant numbers U01/U24 CA074799); Mayo Clinic Cooperative Family Registry for Colon Cancer Studies (grant number U01/U24 CA074800), Ontario Familial Colorectal Cancer Registry (grant number U01/U24 CA074783), Seattle Colorectal Cancer Family Registry (grant number U01/U24 CA074794); University of Hawaii Colorectal Cancer Family Registry (grant number U01/U24 CA074806; National Cancer Institute, National Institutes of Health (NCI/NIH), US Department of Health and Human Services (grant numbers U19 CA148107, R01 CA81488, P30 CA014089, R01 CA197350; P01 CA196569; R01 CA201407); National Institutes of Environmental Health Sciences, National Institutes of Health (grant number T32 ES013678); The American Cancer Society; German Research Council (BR 1704/6‐1, BR 1704/6‐3, BR 1704/6‐4, CH 117/1‐1, HO 5117/2‐1, HE 5998/2‐1, KL 2354/3‐1, RO 2270/8‐1 and BR 1704/17‐1); Interdisciplinary Research Program of the National Center for Tumor Diseases (NCT), Germany; German Federal Ministry of Education and Research (01KH0404, 01ER0814, 01ER0815, 01ER1505A and 01ER1505B); National Institutes of Health (R01 CA48998 to M. L. Slattery); National Institutes of Health (P01 CA055075, UM1 CA167552, U01 CA167552, R01 CA137178, R01 CA151993, and R35 CA197735); National Institutes of Health (R01 CA137178, P01 CA087969, UM1 CA186107, R01 CA151993, and R35 CA197735) and PHS by the National Institutes of Health (R01 CA042182); Clinical Investigator Award from Damon Runyon Cancer Research Foundation (CI‐8); NCI R01CA136726); VicHealth and Cancer Council Victoria; Australian NHMRC grants 509348, 209057, 251553 and 504711; Cancer Council Victoria; National Institutes of Health (R37 CA54281, P01 CA033619, and R01 CA063464); National Institutes of Health, US Department of Health and Human Services (R01 CA81488; Interdisciplinary Health Research Team award from the Canadian Institutes of Health Research (CRT 43821); the National Institutes of Health, US Department of Health and Human Serivces (U01 CA74783); National Cancer Institute of Canada grants (18223 and 18226); Canadian Cancer Society Research Institute; National Institutes of Health, through funding allocated to the Ontario Registry for Studies of Familial Colorectal Cancer (U01 CA074783); Ontario Research Fund, the Canadian Institutes of Health Research, and the Ontario Institute for Cancer Research, through generous support from the Ontario Ministry of Research and Innovation; Intramural Research Program of the Division of Cancer Epidemiology and Genetics and supported by contracts from the Division of Cancer Prevention, National Cancer Institute, NIH, DHHS; National Institutes of Health (NIH), Genes, Environment and Health Initiative (GEI) Z01 CP 010200, NIH U01 HG004446, and NIH GEI U01 HG 004438; NHS in the East of England through the Clinical Academic Reserve. Cancer Research UK (C490/A16561); the UK National Institute for Health Research Biomedical Research Centres at the University of Cambridge; Swedish Research Council/Infrastructure grant, the Swedish Cancer Foundation, and the Karolinska Institute´s Distinguished Professor Award; National Institutes of Health (K05 CA154337); National Heart, Lung, and Blood Institute, National Institutes of Health, US Department of Health and Human Services through contracts HHSN268201100046C, HHSN268201100001C, HHSN268201100002C, HHSN268201100003C, HHSN268201100004C, and HHSN271201100004C; Colorectal Cancer Genetics & Genomics; Instituto de Salud Carlos III, co‐funded by FEDER funds –a way to build Europe– (grants PI14‐613 and PI09‐1286); Agency for Management of University and Research Grants (AGAUR) of the Catalan Government (grant 2017SGR723); Junta de Castilla y León (grant LE22A10‐2); Xarxa de Bancs de Tumors de Catalunya sponsored by Pla Director d’Oncología de Catalunya (XBTC); Plataforma Biobancos PT13/0010/0013 and ICOBIOBANC, sponsored by the Catalan Institute of Oncology; National Institutes of Health (R01 CA076366 and U01 CA074794).

## AUTHOR CONTRIBUTION

ZX, Y‐RS, PP, YL, SB,,XW, LH, PTC, and UP were involved in data analysis and interpretation, JCF, YL, DA, SIB, SB, DDB, GC, ATC, SJG, GGG, SBG, MJG, MH, MAJ, ADJ, LLM, LL, NML, VM, NM, PAN, GR, AW, MOW, HB, EW, MLS, ELG, JC‐C, PDPP, PTC, and UP were involved in recruitment of study participants, data collection (questionnaire, biospecimens and genotyping), and data harmonization, Y‐RS, HS and UP were involved in study design; all authors have been involved in manuscript writing and review.

## Supporting information

Fig S1A

Fig S1B

Fig S2

Fig S3A

Fig S3B

Fig S4

Table S1

Table S2

Table S3A

Table S3B

Supplementary Material

Supplementary Material

## Data Availability

Research data are not shared.
