# Supplementary material for: Functional informed genome‐wide interaction analysis of body mass index, diabetes and colorectal cancer risk
Source: Cancer Med. 2020 Mar 24;9(10):3563–73. doi: 10.1002/cam4.2971 (PMC7221445; doi:10.1002/cam4.2971)

Source	OR (95% CI)
ARCTIC	1.08 [0.85; 1.37]
ATBC	1.29 [0.76; 2.21]
CCFR 1.Australia	1.70 [1.16; 2.47]
CCFR 1.Ontario	1.45 [1.14; 1.85]
CCFR 1.Seattle	1.18 [0.92; 1.51]
CCFR 2.Los Angeles	0.88 [0.57; 1.35]
CCFR 2.Mayo Foundation	1.25 [0.85; 1.83]
CCFR_3	0.95 [0.79; 1.14]
CCFR_4	1.35 [1.14; 1.58]
Colo2&3	2.02 [1.25; 3.28]
CPSII_1	1.32 [1.04; 1.66]
DACHS 1	1.32 [1.15; 1.50]
DACHS 2	1.39 [1.12; 1.73]
DALS 1	1.49 [1.25; 1.76]
DALS 2	1.56 [1.23; 1.97]
HPFS 1	1.48 [1.07; 2.03]
HPFS Ad	1.17 [0.91; 1.51]
Kentucky	1.21 [1.07; 1.36]
MCCS_1	1.29 [1.00; 1.68]
MCCS_2	1.22 [0.84; 1.78]
MEC	1.26 [0.97; 1.64]
MECC_1	1.12 [0.89; 1.42]
MECC_2	1.23 [1.05; 1.44]
MECC_3	1.25 [1.13; 1.39]
NFCCR_2	1.66 [1.25; 2.20]
PHS	1.56 [1.19; 2.04]
PLCO 1 rematch	0.93 [0.76; 1.12]
PLCO 2	1.06 [0.85; 1.32]
SEARCH	1.14 [0.81; 1.59]
SMC_COSM	1.73 [1.19; 2.54]
Spain	0.85 [0.71; 1.01]
VITAL	1.35 [1.04; 1.76]
Total (fixed effect)	1.24 [1.19; 1.29]
Total (random effects)	1.25 [1.18; 1.33]
95% PI	[0.96; 1.64]

Heterogeneity: $\chi^2_{31} = 73.88$ ($P < .01$), $I^2 = 58\%$

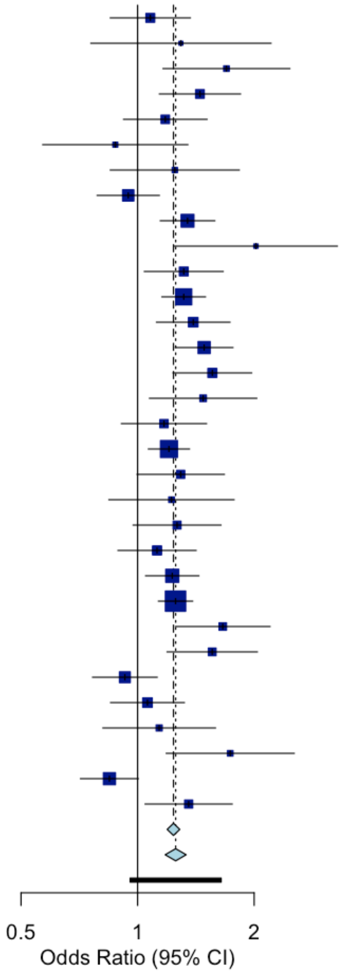

Supplement: Supplementary file 1 — Fig S1A [file CAM4-9-3563-s001.pdf]
