# Supplementary material for: Functional informed genome‐wide interaction analysis of body mass index, diabetes and colorectal cancer risk
Source: Cancer Med. 2020 Mar 24;9(10):3563–73. doi: 10.1002/cam4.2971 (PMC7221445; doi:10.1002/cam4.2971)

**Source**	**OR (95% CI)**
ARCTIC	1.04 [0.86; 1.27]
CCFR 1.Australia	1.16 [0.93; 1.45]
CCFR 1.Ontario	0.96 [0.80; 1.15]
CCFR 1.Seattle	1.13 [0.95; 1.34]
CCFR 2.Los Angeles	0.82 [0.58; 1.14]
CCFR 2.Mayo Foundation	0.94 [0.69; 1.26]
CCFR_3	1.12 [0.99; 1.26]
CCFR_4	1.41 [1.21; 1.64]
Colo2&3	0.93 [0.60; 1.43]
CPSII_1	1.28 [1.06; 1.54]
DACHS 1	1.25 [1.11; 1.42]
DACHS 2	1.44 [1.13; 1.83]
DALS 1	1.24 [1.06; 1.45]
DALS 2	1.14 [0.96; 1.37]
Kentucky	1.16 [1.05; 1.28]
MCCS_1	1.12 [0.91; 1.37]
MCCS_2	1.06 [0.80; 1.38]
MEC	1.19 [0.95; 1.48]
MECC_1	1.02 [0.84; 1.25]
MECC_2	1.14 [1.00; 1.29]
MECC_3	0.94 [0.86; 1.03]
NFCCR_2	1.29 [1.00; 1.68]
NHS 1	1.00 [0.85; 1.17]
NHS Ad	1.23 [1.06; 1.41]
PLCO 1 rematch	1.37 [1.11; 1.68]
PLCO 2	1.02 [0.82; 1.26]
PMH-SCCFR	1.40 [1.12; 1.75]
SEARCH	1.07 [0.78; 1.48]
SMC_COSM	1.09 [0.86; 1.37]
Spain	0.88 [0.73; 1.05]
VITAL	1.24 [0.98; 1.58]
WHI 1 rematch	1.27 [1.12; 1.44]
WHI 2	1.04 [0.96; 1.13]
Total (fixed effect)	1.12 [1.09; 1.15]
Total (random effects)	1.13 [1.08; 1.18]
95% PI	[0.93; 1.38]

Heterogeneity: χ^2_{32} = 74.30 (P < .01), I^2 = 57%

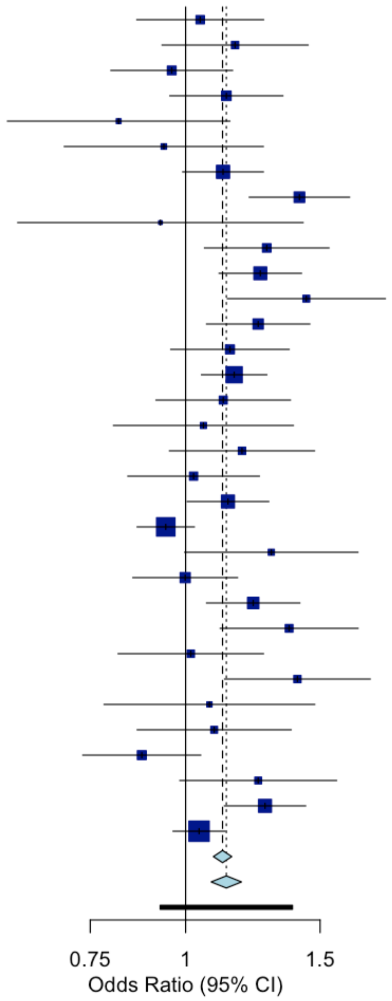

Supplement: Supplementary file 2 — Fig S1B [file CAM4-9-3563-s002.pdf]
