# Supplementary material for: Functional informed genome‐wide interaction analysis of body mass index, diabetes and colorectal cancer risk
Source: Cancer Med. 2020 Mar 24;9(10):3563–73. doi: 10.1002/cam4.2971 (PMC7221445; doi:10.1002/cam4.2971)

Source	OR (95% CI)
ASTERISK	2.46 [1.72; 3.52]
CCFR 1.Australia	1.94 [0.73; 5.17]
CCFR 1.Ontario	0.82 [0.50; 1.33]
CCFR 1.Seattle	0.88 [0.53; 1.45]
CCFR 2.Los Angeles	0.94 [0.32; 2.75]
CCFR 2.Mayo Foundation	1.24 [0.42; 3.68]
CCFR_3	0.95 [0.69; 1.32]
CCFR_4	3.01 [1.93; 4.71]
Colo2&3	1.23 [0.40; 3.79]
CPSII_1	1.12 [0.71; 1.75]
DACHS 1	1.38 [1.15; 1.66]
DACHS 2	1.35 [0.98; 1.84]
HPFS 1	8.19 [0.98; 68.68]
HPFS Ad	0.55 [0.05; 6.05]
Kentucky	1.30 [1.04; 1.62]
MCCS_1	1.61 [0.79; 3.29]
MCCS_2	1.97 [0.58; 6.65]
MEC	1.12 [0.59; 2.15]
MECC_1	0.96 [0.70; 1.31]
MECC_2	1.07 [0.87; 1.33]
MECC_3	0.87 [0.75; 1.00]
NFCCR_2	2.01 [1.30; 3.10]
NHS 1	2.20 [1.07; 4.51]
NHS Ad	1.78 [0.69; 4.64]
PHS	1.04 [0.44; 2.44]
PLCO 1 rematch	1.33 [0.84; 2.11]
PLCO 2	1.64 [0.96; 2.80]
PMH-SCCFR	1.63 [0.85; 3.15]
SMC_COSM	1.60 [1.12; 2.28]
Spain	1.11 [0.84; 1.47]
VITAL	5.30 [1.15; 24.40]
WHI 1 rematch	2.11 [1.17; 3.82]
WHI 2	1.51 [1.03; 2.19]
Total (fixed effect)	1.22 [1.14; 1.30]
Total (random effects)	1.37 [1.20; 1.56]
95% PI	[0.78; 2.40]

Heterogeneity: $\chi^2_{32} = 92.43$ ($P < .01$), $I^2 = 65\%$

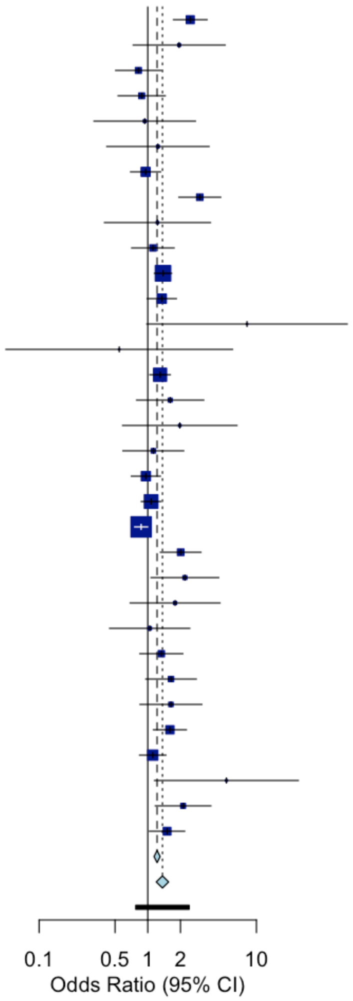

Supplement: Supplementary file 3 — Fig S2 [file CAM4-9-3563-s003.pdf]
