# Supplementary figures and images for: Functional informed genome‐wide interaction analysis of body mass index, diabetes and colorectal cancer risk
Source: Cancer Med. 2020 Mar 24;9(10):3563–73. doi: 10.1002/cam4.2971 (PMC7221445; doi:10.1002/cam4.2971)

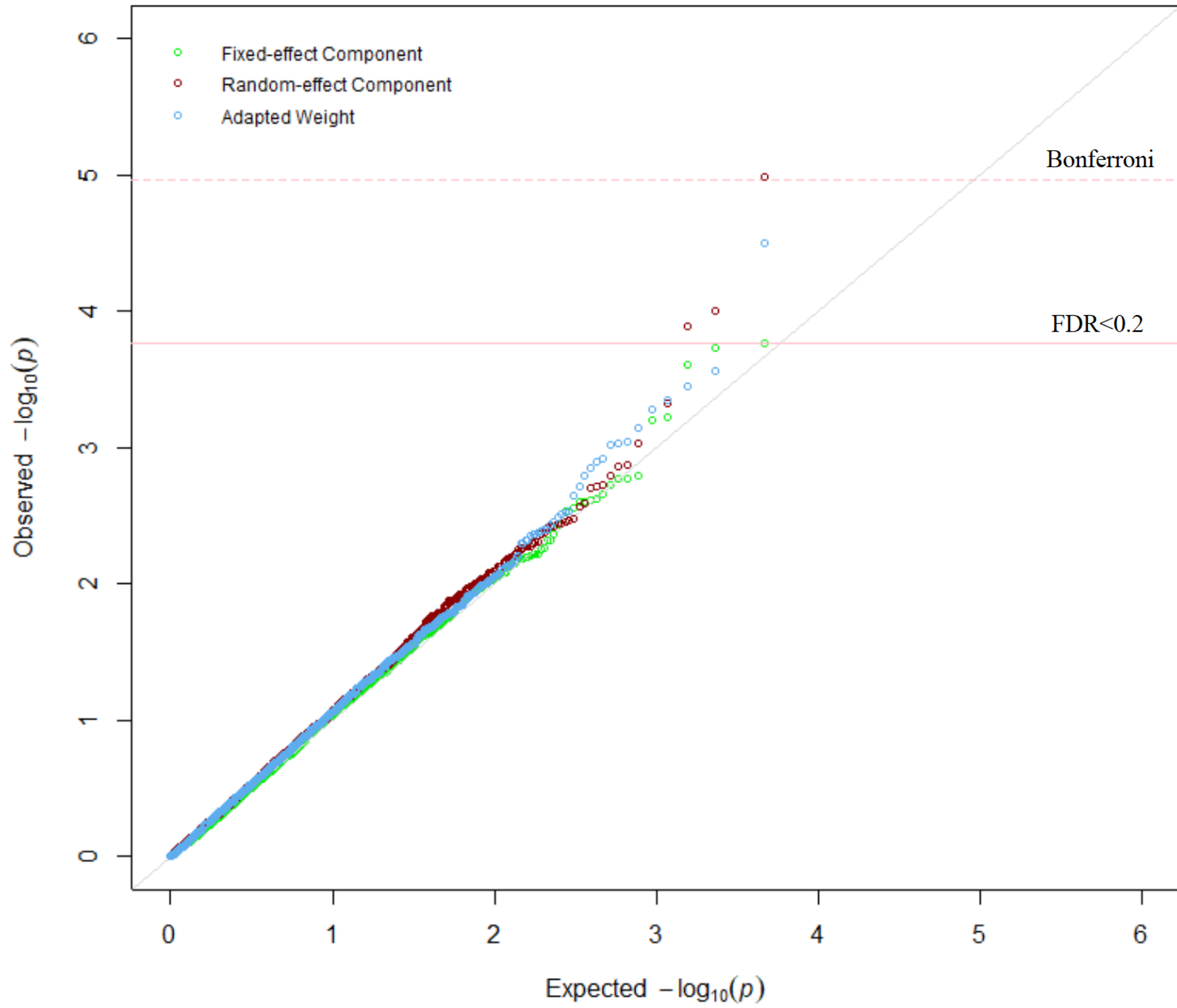

Supplement: Supplementary file 4 — Fig S3A [file CAM4-9-3563-s004.pdf]

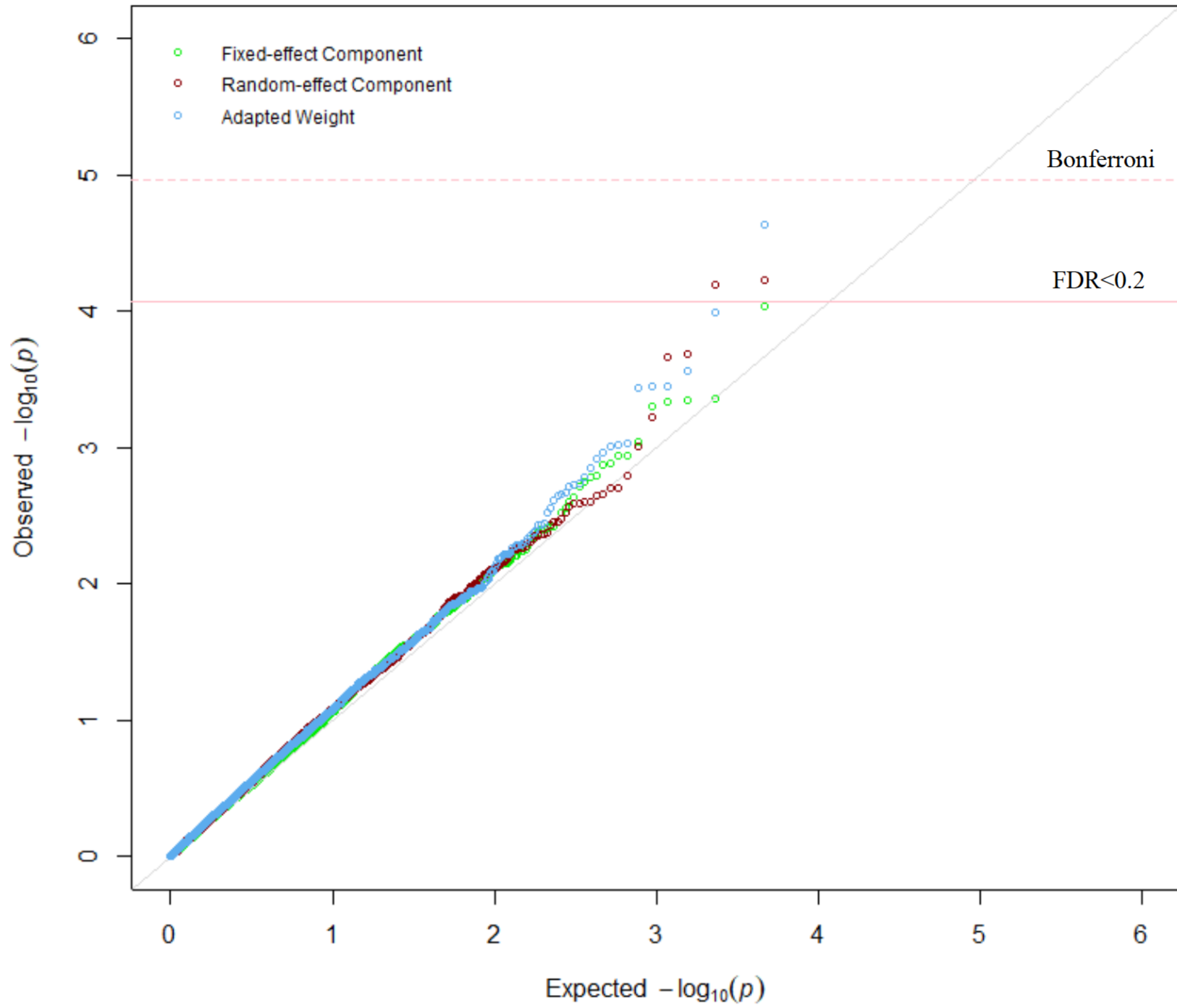

Supplement: Supplementary file 5 — Fig S3B [file CAM4-9-3563-s005.pdf]

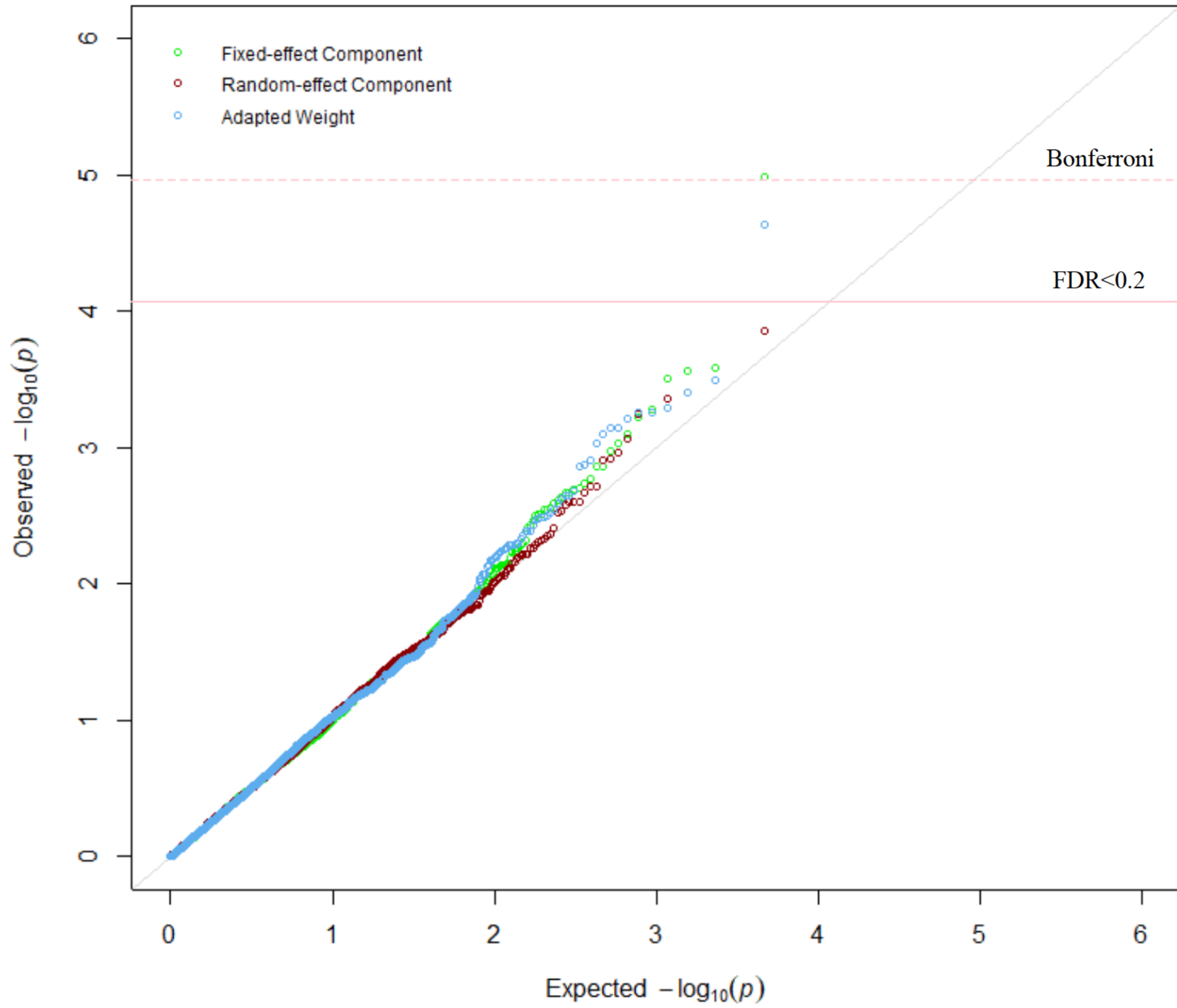

Supplement: Supplementary file 6 — Fig S4 [file CAM4-9-3563-s006.pdf]
