# Supplementary material for: Functional informed genome‐wide interaction analysis of body mass index, diabetes and colorectal cancer risk
Source: Cancer Med. 2020 Mar 24;9(10):3563–73. doi: 10.1002/cam4.2971 (PMC7221445; doi:10.1002/cam4.2971)
Supplement: Supplementary file 7 — Table S1 [file CAM4-9-3563-s007.docx]

**Supplementary Table 1. Demographic characteristics of study participants (N = 46,709)**

|  | **Cases**  **(n = 26,017)** | **Controls**  **(n = 20,692)** |
| --- | --- | --- |
| **Age (years, mean±SD)** | 63.9±10.8 | 64.2±10.7 |
| **Sex (%)** |  |  |
| Male | 13207 (50.7) | 9861 (47.6) |
| Female | 12817 (49.3) | 10834 (52.4) |
| **BMI (kg/m^2^, mean±SD)** | 27.4±4.9 | 26.7±4.6 |
| **Type 2 diabetes (%)** |  |  |
| Yes | 2611 (13.5) | 1999 (10.7) |
| No | 16771 (86.5) | 16620 (89.3) |
