# Supplementary material for: Functional informed genome‐wide interaction analysis of body mass index, diabetes and colorectal cancer risk
Source: Cancer Med. 2020 Mar 24;9(10):3563–73. doi: 10.1002/cam4.2971 (PMC7221445; doi:10.1002/cam4.2971)
Supplement: Supplementary file 8 — Table S2 [file CAM4-9-3563-s008.docx]

**Supplementary Table 2. Multivariable generalized regression analyses on main effects and interactions between individual genetic variants and BMI/Diabetes on CRC risk for each gene set listed in Table 1 (most significant genetic variants only)**

| **Male – BMI** | | | | | | | | | | |
| --- | --- | --- | --- | --- | --- | --- | --- | --- | --- | --- |
| Gene | rsid | SNPs | | MAF | Main effect | | | Interaction with BMI^1^ | | |
|  |  |  | |  | Coefficient | SE | P-value | Coefficient | SE | P-value |
| ***FOXA1*** |  |  | |  |  |  |  |  |  |  |
|  | rs17105308 | 14:37255137_T/C | | 0.213808 | 0.704 | 0.174 | **0.000053** | -0.124 | 0.032 | **0.000097** |
|  | rs17105347 | 14:37265451_T/C | | 0.214051 | 0.919 | 0.413 | **0.026030** | -0.124 | 0.032 | **0.000108** |
|  | rs17106861 | 14:38048983_A/G | | 0.085596 | 0.606 | 0.253 | **0.016425** | -0.111 | 0.046 | **0.015417** |
|  | rs74539758 | 14:38644887_T/C | | 0.214186 | 0.382 | 0.175 | **0.028802** | -0.075 | 0.032 | **0.017801** |
|  | rs8020485 | 14:38954931_C/T | | 0.170332 | 0.389 | 0.194 | **0.045158** | -0.076 | 0.035 | **0.032935** |
|  | rs78072102 | 14:38035740_T/C | | 0.025710 | -1.062 | 0.533 | **0.046408** | 0.194 | 0.098 | **0.046668** |
|  | rs409755 | 14:37325761_C/T | | 0.300672 | -0.323 | 0.161 | **0.045346** | 0.056 | 0.029 | 0.057056 |
| ***CD33*** |  |  | |  |  |  |  |  |  |  |
|  | rs1973095 | 19:52003864_C/T | | 0.183584 | -0.572 | 0.185 | **0.002031** | 0.100 | 0.034 | **0.003009** |
|  | rs1673028 | 19:50953053_T/C | | 0.324537 | -0.356 | 0.157 | **0.022868** | 0.068 | 0.029 | **0.017965** |
|  | rs55744949 | 19:51762218_C/T | | 0.040029 | 0.872 | 0.375 | **0.020158** | -0.153 | 0.068 | **0.024189** |
|  | rs3810113 | 19:52006071_A/G | | 0.183386 | -0.523 | 0.228 | **0.021611** | 0.085 | 0.033 | **0.011512** |
|  | rs1551555 | 19:52005334_G/A | | 0.183649 | -0.889 | 0.691 | 0.198479 | 0.084 | 0.034 | **0.012165** |
| ***PSMC5*** |  |  | |  |  |  |  |  |  |  |
|  | rs7225568 | 17:61611423_T/C | | 0.385331 | -0.608 | 0.150 | **0.000050** | 0.109 | 0.027 | **0.000072** |
|  | rs12937836 | 17:61609510_A/G | | 0.376181 | -0.551 | 0.168 | **0.000999** | 0.096 | 0.027 | **0.000430** |
|  | rs12939133 | 17:62077851_G/A | | 0.432152 | 0.485 | 0.164 | **0.003116** | -0.082 | 0.030 | **0.006430** |
|  | rs73328128 | 17:62277045_C/T | | 0.047991 | 0.957 | 0.380 | **0.011874** | -0.192 | 0.070 | **0.006137** |
|  | rs12939821 | 17:62079286_G/A | | 0.421491 | 0.367 | 0.173 | **0.034076** | -0.068 | 0.030 | **0.023418** |
|  | rs111735595 | 17:61828142_T/C | | 0.060752 | -0.834 | 0.366 | **0.022636** | 0.161 | 0.067 | **0.016636** |
|  | rs9901723 | 17:62244844_A/G | | 0.048795 | 0.719 | 0.415 | 0.083244 | -0.178 | 0.068 | **0.009429** |
|  | | | | | | | | | | |
| **Female – BMI** | | | | | | | | | | |
| Gene | rsid | SNPs | | MAF | Main effect | | | Interaction with BMI | | |
|  |  |  | |  | Beta | SE | P-value | Beta | SE | P-value |
| ***KIAA0753*** |  |  | |  |  |  |  |  |  |  |
|  | rs4796407 | 17:7245371_A/G | | 0.415476 | 0.290 | 0.121 | **0.016313** | -0.050 | 0.022 | **0.023539** |
|  | rs76018634 | 17:6732660_G/A | | 0.061188 | -0.518 | 0.243 | **0.033116** | 0.108 | 0.045 | **0.015002** |
|  | rs34986335 | 17:6489231_C/T | | 0.154432 | 0.352 | 0.149 | **0.017899** | -0.061 | 0.027 | **0.025080** |
|  | rs4465647 | 17:7244477_A/G | | 0.359115 | 0.314 | 0.133 | **0.018221** | -0.050 | 0.024 | **0.041482** |
| ***SCN1B*** |  |  | |  |  |  |  |  |  |  |
|  | rs4806092 | 19:35623589_A/G | | 0.463010 | 0.437 | 0.113 | **0.000112** | -0.086 | 0.021 | **0.000037** |
|  | rs11666576 | 19:35624890_C/T | | 0.465139 | 0.755 | 0.315 | **0.016578** | -0.084 | 0.021 | **0.000046** |
|  | | | | | | | | | | |
| **Diabetes** | | | | | | | | | | |
| Gene | rsid | SNPs | | MAF | Main effect | | | Interaction with Diabetes | | |
|  |  |  | |  | Coefficient | SE | P-value | Coefficient | SE | P-value |
| ***PTPN2*** | | | | | | | | | | |
|  | rs34744608 | | 18:13809142_C/T | 0.063468 | 0.045 | 0.034 | 0.181352 | -0.359 | 0.092 | **0.000103** |
|  | rs9959699 | | 18:13428038_T/C | 0.485715 | 0.010 | 0.016 | 0.51776 | 0.111 | 0.048 | **0.02020** |
|  | rs12957151 | | 18:12576001_A/G | 0.440878 | 0.023 | 0.015 | 0.14298 | 0.098 | 0.046 | **0.03278** |
|  | rs28496036 | | 18:12579282_T/C | 0.441151 | 0.301 | 0.205 | 0.14131 | -0.094 | 0.046 | **0.04051** |

Note: A sequential analysis was conducted, where we started with the most significant SNP with the GxE interaction, then took the next significant one while adjusting for the first one, and so forth until the SNP’s p-value was greater than 0.05.
