# Supplementary material for: Functional informed genome‐wide interaction analysis of body mass index, diabetes and colorectal cancer risk
Source: Cancer Med. 2020 Mar 24;9(10):3563–73. doi: 10.1002/cam4.2971 (PMC7221445; doi:10.1002/cam4.2971)
Supplement: Supplementary file 9 — Table S3A [file CAM4-9-3563-s009.docx]

**Supplementary Table 3a. The association of the predicted gene expression on CRC risk stratified by sex and BMI at quartiles**

|  | **1^st^ quartile** | | **2^nd^ quartile** | | **3^rd^ quartile** | | **4^th^ quartile** | |
| --- | --- | --- | --- | --- | --- | --- | --- | --- |
|  | OR | 95% CI | OR | 95% CI | OR | 95% CI | OR | 95% CI |
| **Male** |  |  |  |  |  | |  | |
| ***FOXA1*** | 0.724 | 0.532 - 0.985 | 1.004 | 0.739 - 1.365 | 1.006 | 0.745 - 1.358 | 1.224 | 0.892 - 1.679 |
| ***CD33*** | 1.015 | 0.815 - 1.263 | 1.049 | 0.838 - 1.313 | 1.157 | 0.928 - 1.442 | 1.196 | 0.958 - 1.492 |
| ***PSMC5*** | 1.017 | 0.724 - 1.431 | 1.084 | 0.779 - 1.507 | 1.190 | 0.861 - 1.644 | 1.214 | 0.866 - 1.702 |
| **Female** |  |  |  |  |  |  |  |  |
| ***KIAA0753*** | 0.928 | 0.776 - 1.110 | 0.950 | 0.797 - 1.133 | 1.075 | 0.901 - 1.283 | 1.137 | 0.951 - 1.360 |
| ***SCN1B*** | 0.759 | 0.448 - 1.295 | 0.823 | 0.477 - 1.421 | 1.140 | 0.673 - 1.934 | 1.293 | 0.767 - 2.179 |
