# Supplementary material for: Functional informed genome‐wide interaction analysis of body mass index, diabetes and colorectal cancer risk
Source: Cancer Med. 2020 Mar 24;9(10):3563–73. doi: 10.1002/cam4.2971 (PMC7221445; doi:10.1002/cam4.2971)
Supplement: Supplementary file 10 — Table S3B [file CAM4-9-3563-s010.docx]

**Supplementary Table 3b. The association of the predicted gene expression on CRC risk stratified by diabetes**

|  | **Non-diabetes** | | **Diabetes** | |
| --- | --- | --- | --- | --- |
|  | OR | 95% CI | OR | 95% CI |
| ***PTPN2*** | 1.031 | 0.975 - 1.091 | 1.200 | 1.027 - 1.401 |
