# Supplementary material for: Functional informed genome‐wide interaction analysis of body mass index, diabetes and colorectal cancer risk
Source: Cancer Med. 2020 Mar 24;9(10):3563–73. doi: 10.1002/cam4.2971 (PMC7221445; doi:10.1002/cam4.2971)

**Figure Legends**

Supplementary Figure 1a. Main effect of BMI in men (per 5 kg/m^2^)

Supplementary Figure 1b. Main effect of BMI in women (per 5 kg/m^2^)

Supplementary Figure 2. Main effect of Diabetes

Supplementary Figure 3a. Quantile-quantile plot of p-values of GxBMI in men

Supplementary Figure 3b. Quantile-quantile plot of p-values of GxBMI in women

Supplementary Figure 4. Quantile-quantile plot of p-values of GxDiabetes

**Supplementary Table 1.** Demographic characteristics of study participants (N = 46,709)

|  | **Cases**  **(n = 26,017)** | **Controls**  **(n = 20,692)** |
| --- | --- | --- |
| **Age (years, mean±SD)** | 63.9±10.8 | 64.2±10.7 |
| **Sex (%)** |  |  |
| Male | 13207 (50.7) | 9861 (47.6) |
| Female | 12817 (49.3) | 10834 (52.4) |
| **BMI (kg/m^2^, mean±SD)** | 27.4±4.9 | 26.7±4.6 |
| **Type 2 diabetes (%)** |  |  |
| Yes | 2611 (13.5) | 1999 (10.7) |
| No | 16771 (86.5) | 16620 (89.3) |

**Supplementary Table 2. Multivariable generalized regression analyses on main effects and interactions between individual genetic variants and BMI/Diabetes on CRC risk for each gene set listed in Table 1 (most significant genetic variants only)**

| **Male – BMI** | | | | | | | | | | |
| --- | --- | --- | --- | --- | --- | --- | --- | --- | --- | --- |
| Gene | rsid | SNPs | | MAF | Main effect | | | Interaction with BMI^1^ | | |
|  |  |  | |  | Coefficient | SE | P-value | Coefficient | SE | P-value |
| ***FOXA1*** |  |  | |  |  |  |  |  |  |  |
|  | rs17105308 | 14:37255137_T/C | | 0.213808 | 0.704 | 0.174 | **0.000053** | -0.124 | 0.032 | **0.000097** |
|  | rs17105347 | 14:37265451_T/C | | 0.214051 | 0.919 | 0.413 | **0.026030** | -0.124 | 0.032 | **0.000108** |
|  | rs17106861 | 14:38048983_A/G | | 0.085596 | 0.606 | 0.253 | **0.016425** | -0.111 | 0.046 | **0.015417** |
|  | rs74539758 | 14:38644887_T/C | | 0.214186 | 0.382 | 0.175 | **0.028802** | -0.075 | 0.032 | **0.017801** |
|  | rs8020485 | 14:38954931_C/T | | 0.170332 | 0.389 | 0.194 | **0.045158** | -0.076 | 0.035 | **0.032935** |
|  | rs78072102 | 14:38035740_T/C | | 0.025710 | -1.062 | 0.533 | **0.046408** | 0.194 | 0.098 | **0.046668** |
|  | rs409755 | 14:37325761_C/T | | 0.300672 | -0.323 | 0.161 | **0.045346** | 0.056 | 0.029 | 0.057056 |
| ***CD33*** |  |  | |  |  |  |  |  |  |  |
|  | rs1973095 | 19:52003864_C/T | | 0.183584 | -0.572 | 0.185 | **0.002031** | 0.100 | 0.034 | **0.003009** |
|  | rs1673028 | 19:50953053_T/C | | 0.324537 | -0.356 | 0.157 | **0.022868** | 0.068 | 0.029 | **0.017965** |
|  | rs55744949 | 19:51762218_C/T | | 0.040029 | 0.872 | 0.375 | **0.020158** | -0.153 | 0.068 | **0.024189** |
|  | rs3810113 | 19:52006071_A/G | | 0.183386 | -0.523 | 0.228 | **0.021611** | 0.085 | 0.033 | **0.011512** |
|  | rs1551555 | 19:52005334_G/A | | 0.183649 | -0.889 | 0.691 | 0.198479 | 0.084 | 0.034 | **0.012165** |
| ***PSMC5*** |  |  | |  |  |  |  |  |  |  |
|  | rs7225568 | 17:61611423_T/C | | 0.385331 | -0.608 | 0.150 | **0.000050** | 0.109 | 0.027 | **0.000072** |
|  | rs12937836 | 17:61609510_A/G | | 0.376181 | -0.551 | 0.168 | **0.000999** | 0.096 | 0.027 | **0.000430** |
|  | rs12939133 | 17:62077851_G/A | | 0.432152 | 0.485 | 0.164 | **0.003116** | -0.082 | 0.030 | **0.006430** |
|  | rs73328128 | 17:62277045_C/T | | 0.047991 | 0.957 | 0.380 | **0.011874** | -0.192 | 0.070 | **0.006137** |
|  | rs12939821 | 17:62079286_G/A | | 0.421491 | 0.367 | 0.173 | **0.034076** | -0.068 | 0.030 | **0.023418** |
|  | rs111735595 | 17:61828142_T/C | | 0.060752 | -0.834 | 0.366 | **0.022636** | 0.161 | 0.067 | **0.016636** |
|  | rs9901723 | 17:62244844_A/G | | 0.048795 | 0.719 | 0.415 | 0.083244 | -0.178 | 0.068 | **0.009429** |
|  | | | | | | | | | | |
| **Female – BMI** | | | | | | | | | | |
| Gene | rsid | SNPs | | MAF | Main effect | | | Interaction with BMI | | |
|  |  |  | |  | Beta | SE | P-value | Beta | SE | P-value |
| ***KIAA0753*** |  |  | |  |  |  |  |  |  |  |
|  | rs4796407 | 17:7245371_A/G | | 0.415476 | 0.290 | 0.121 | **0.016313** | -0.050 | 0.022 | **0.023539** |
|  | rs76018634 | 17:6732660_G/A | | 0.061188 | -0.518 | 0.243 | **0.033116** | 0.108 | 0.045 | **0.015002** |
|  | rs34986335 | 17:6489231_C/T | | 0.154432 | 0.352 | 0.149 | **0.017899** | -0.061 | 0.027 | **0.025080** |
|  | rs4465647 | 17:7244477_A/G | | 0.359115 | 0.314 | 0.133 | **0.018221** | -0.050 | 0.024 | **0.041482** |
| ***SCN1B*** |  |  | |  |  |  |  |  |  |  |
|  | rs4806092 | 19:35623589_A/G | | 0.463010 | 0.437 | 0.113 | **0.000112** | -0.086 | 0.021 | **0.000037** |
|  | rs11666576 | 19:35624890_C/T | | 0.465139 | 0.755 | 0.315 | **0.016578** | -0.084 | 0.021 | **0.000046** |
|  | | | | | | | | | | |
| **Diabetes** | | | | | | | | | | |
| Gene | rsid | SNPs | | MAF | Main effect | | | Interaction with Diabetes | | |
|  |  |  | |  | Coefficient | SE | P-value | Coefficient | SE | P-value |
| ***PTPN2*** | | | | | | | | | | |
|  | rs34744608 | | 18:13809142_C/T | 0.063468 | 0.045 | 0.034 | 0.181352 | -0.359 | 0.092 | **0.000103** |
|  | rs9959699 | | 18:13428038_T/C | 0.485715 | 0.010 | 0.016 | 0.51776 | 0.111 | 0.048 | **0.02020** |
|  | rs12957151 | | 18:12576001_A/G | 0.440878 | 0.023 | 0.015 | 0.14298 | 0.098 | 0.046 | **0.03278** |
|  | rs28496036 | | 18:12579282_T/C | 0.441151 | 0.301 | 0.205 | 0.14131 | -0.094 | 0.046 | **0.04051** |

Note: A sequential analysis was conducted, where we started with the most significant SNP with the GxE interaction, then took the next significant one while adjusting for the first one, and so forth until the SNP’s p-value was greater than 0.05.

**Supplementary Table 3a. The association of the predicted gene expression on CRC risk stratified by sex and BMI at quartiles**

|  | **1^st^ quartile** | | **2^nd^ quartile** | | **3^rd^ quartile** | | **4^th^ quartile** | |
| --- | --- | --- | --- | --- | --- | --- | --- | --- |
|  | OR | 95% CI | OR | 95% CI | OR | 95% CI | OR | 95% CI |
| **Male** |  |  |  |  |  | |  | |
| ***FOXA1*** | 0.724 | 0.532 - 0.985 | 1.004 | 0.739 - 1.365 | 1.006 | 0.745 - 1.358 | 1.224 | 0.892 - 1.679 |
| ***CD33*** | 1.015 | 0.815 - 1.263 | 1.049 | 0.838 - 1.313 | 1.157 | 0.928 - 1.442 | 1.196 | 0.958 - 1.492 |
| ***PSMC5*** | 1.017 | 0.724 - 1.431 | 1.084 | 0.779 - 1.507 | 1.190 | 0.861 - 1.644 | 1.214 | 0.866 - 1.702 |
| **Female** |  |  |  |  |  |  |  |  |
| ***KIAA0753*** | 0.928 | 0.776 - 1.110 | 0.950 | 0.797 - 1.133 | 1.075 | 0.901 - 1.283 | 1.137 | 0.951 - 1.360 |
| ***SCN1B*** | 0.759 | 0.448 - 1.295 | 0.823 | 0.477 - 1.421 | 1.140 | 0.673 - 1.934 | 1.293 | 0.767 - 2.179 |

**Supplementary Table 3b. The association of the predicted gene expression on CRC risk stratified by diabetes**

|  | **Non-diabetes** | | **Diabetes** | |
| --- | --- | --- | --- | --- |
|  | OR | 95% CI | OR | 95% CI |
| ***PTPN2*** | 1.031 | 0.975 - 1.091 | 1.200 | 1.027 - 1.401 |

**Supplementary Figure 1a. Main effect of BMI in men (per 5 kg/m^2^)**

**
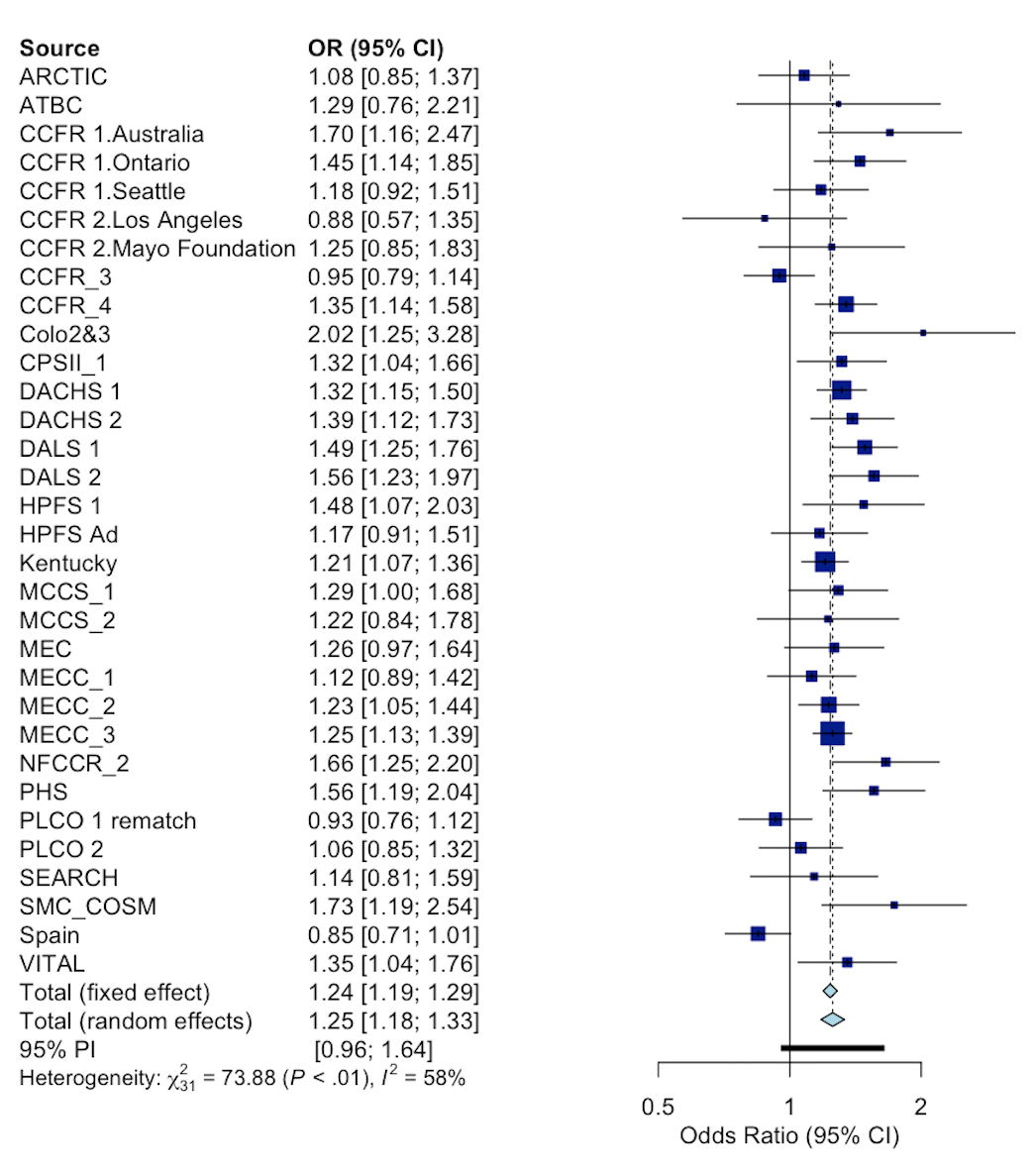
**

**Supplementary Figure 1b. Main effect of BMI in women (per 5 kg/m^2^)**

**
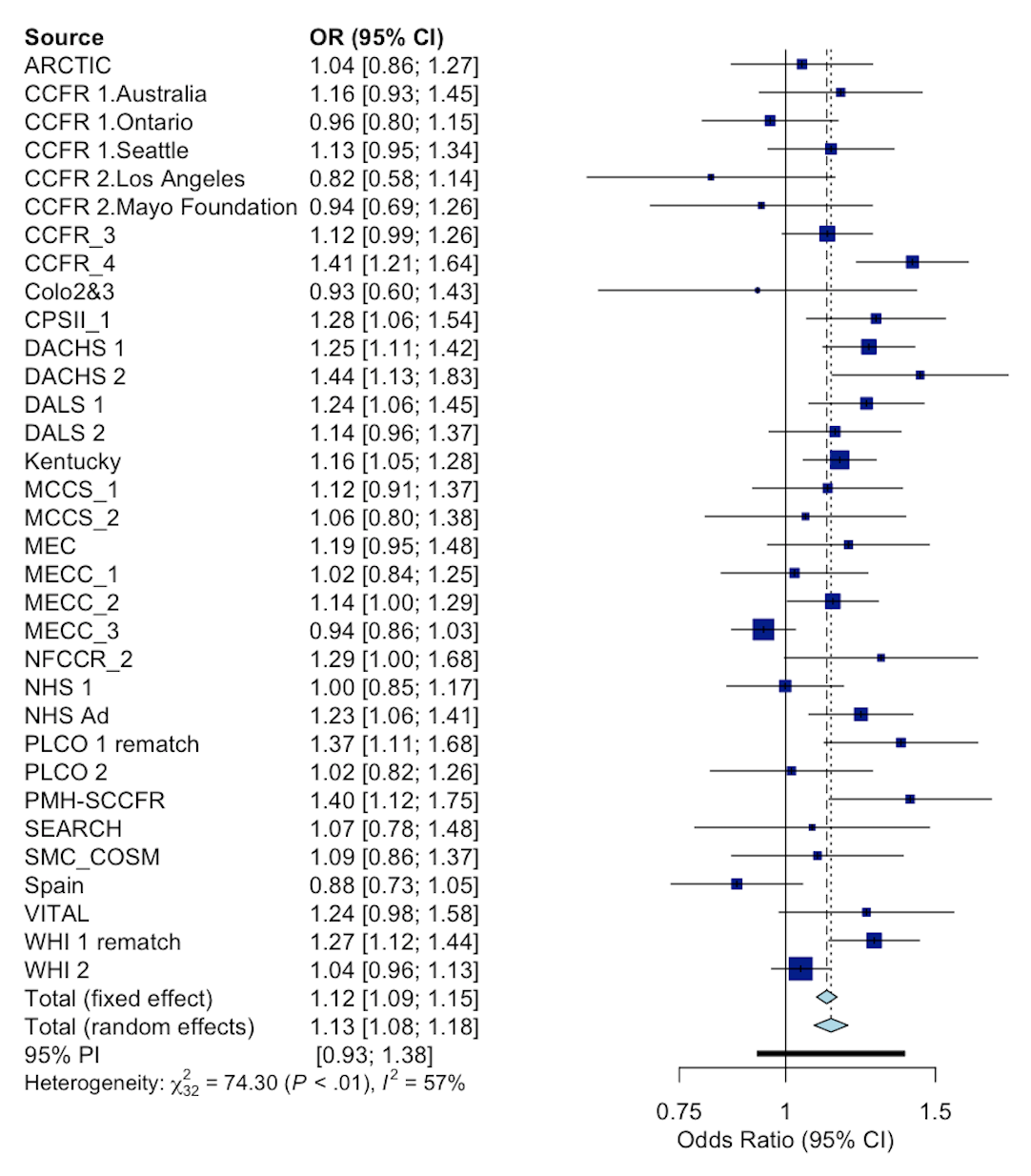
**

**Supplementary Figure 2. Main effect of Diabetes**

**
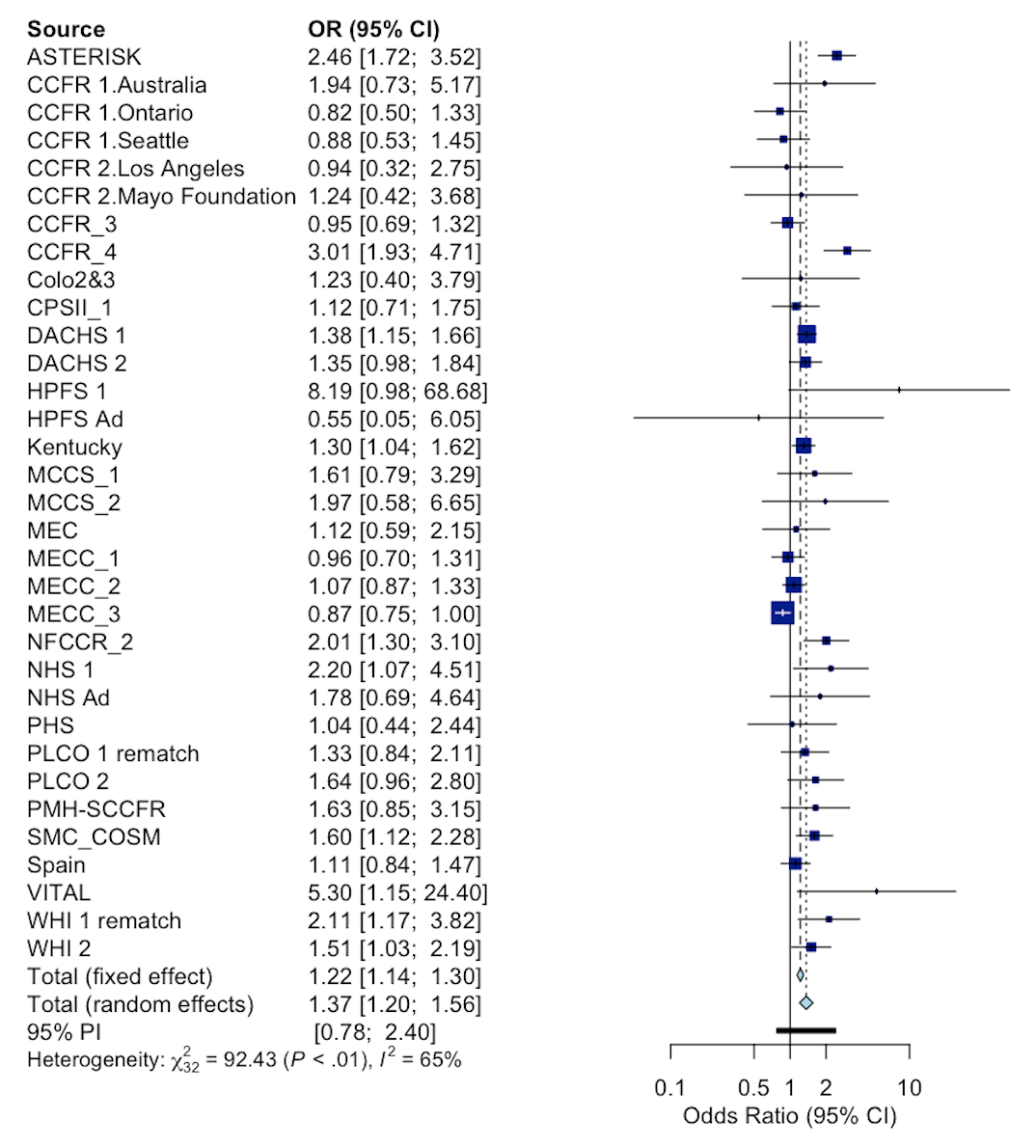
**

**Supplementary Figure 3a. Quantile-quantile plot of p-values of GxBMI in men**


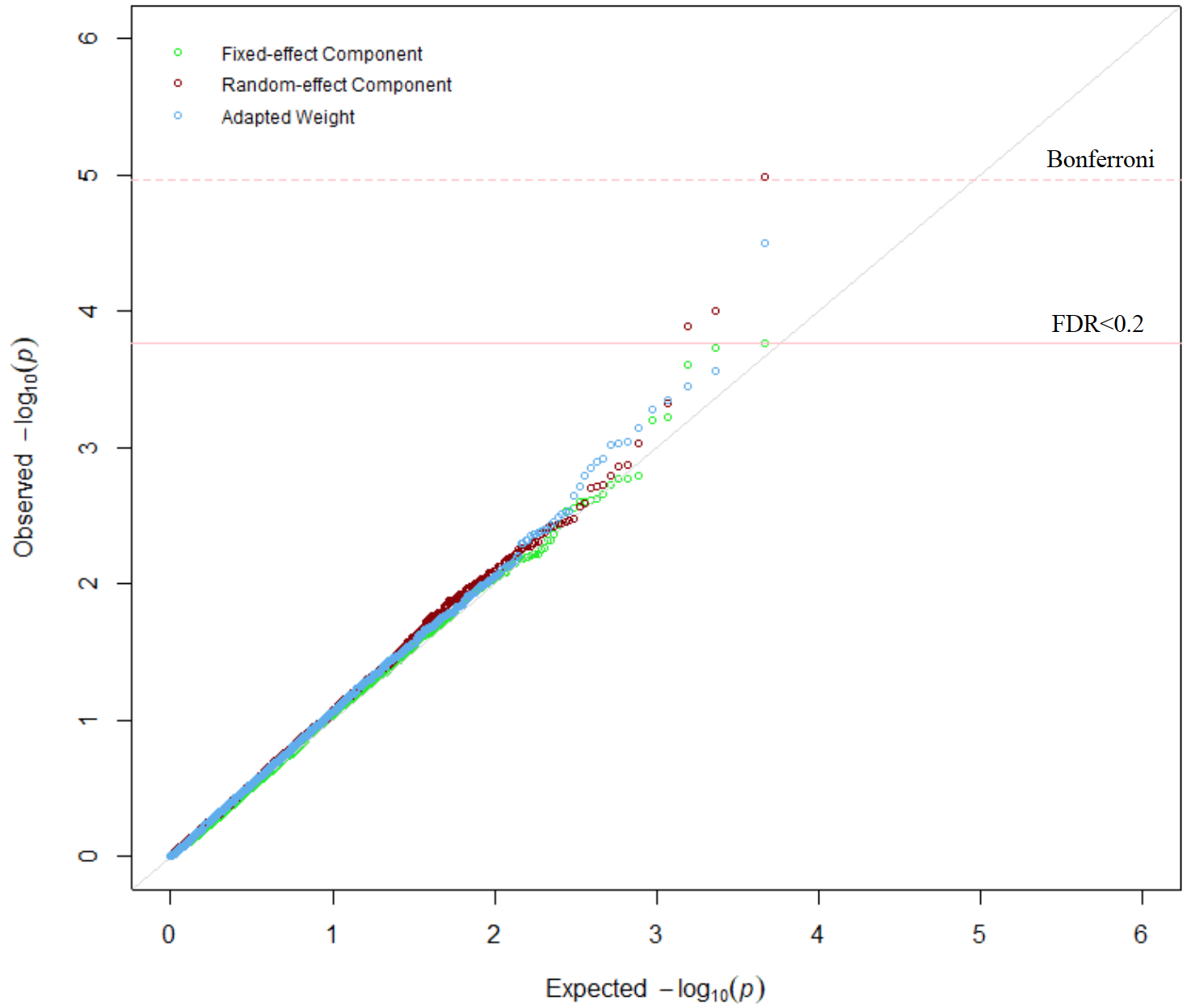


**Supplementary Figure 3b. Quantile-quantile plot of p-values of GxBMI in women**


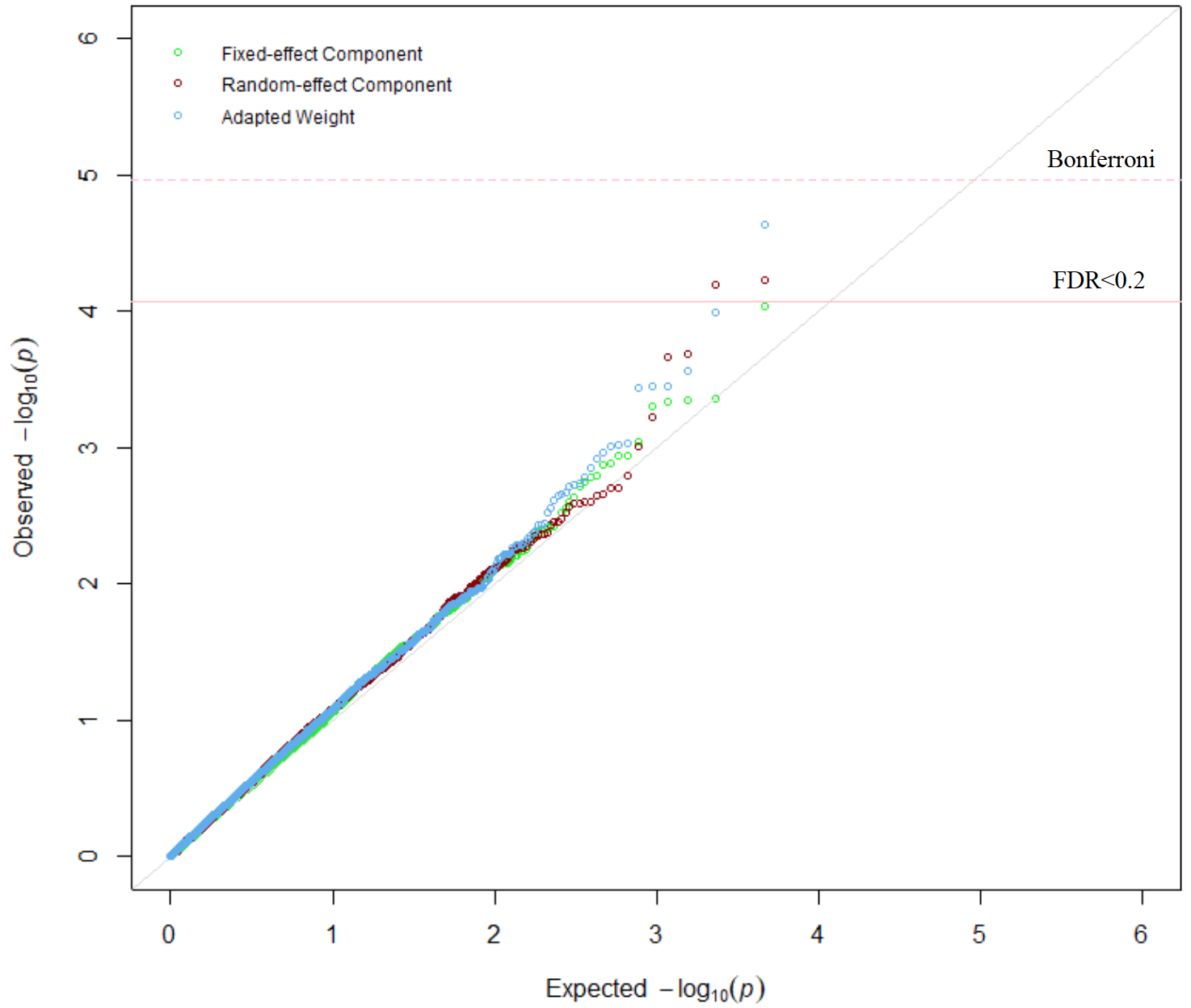


**Supplementary Figure 4. Quantile-quantile plot of p-values of GxDiabetes**


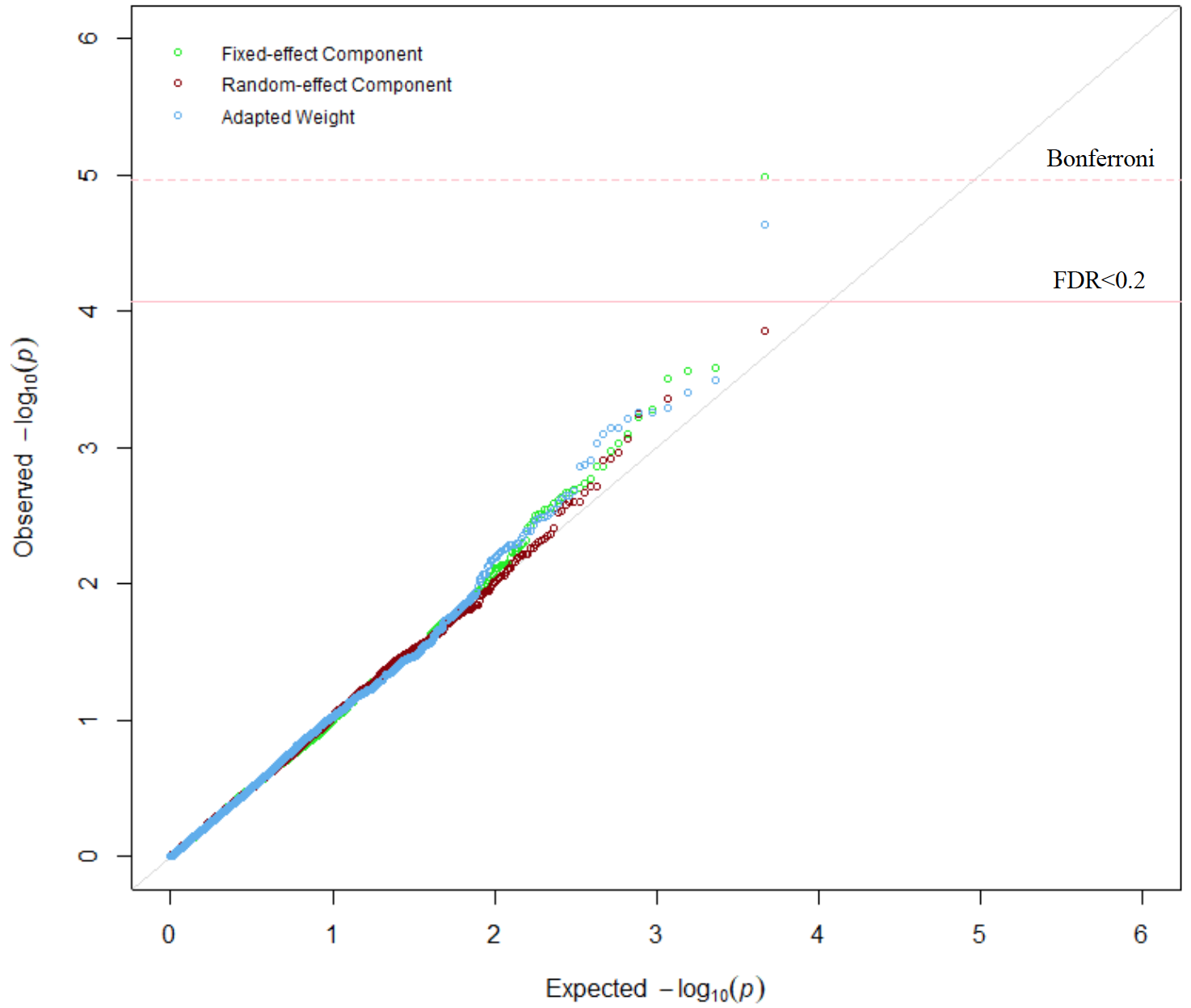

Supplement: Supplementary file 11 — Supplementary Material [file CAM4-9-3563-s011.doc]
