# Supplementary material for: Functional informed genome‐wide interaction analysis of body mass index, diabetes and colorectal cancer risk
Source: Cancer Med. 2020 Mar 24;9(10):3563–73. doi: 10.1002/cam4.2971 (PMC7221445; doi:10.1002/cam4.2971)
Supplement: Supplementary file 12 — Supplementary Material [file CAM4-9-3563-s012.pdf]

Figure Legends

Supplementary Figure 1a. Main effect of BMI in men (per 5 kg/m^2)

Supplementary Figure 1b. Main effect of BMI in women (per 5 kg/m^2)

Supplementary Figure 2. Main effect of Diabetes

Supplementary Figure 3a. Quantile-quantile plot of p-values of GxBMI in men

Supplementary Figure 3b. Quantile-quantile plot of p-values of GxBMI in women

Supplementary Figure 4. Quantile-quantile plot of p-values of GxDiabetes
